# A Part of the Medial Branch of the Deep Peroneal Nerve Distributes the Dorsal Pedis Artery and Its Distribution Area is Close to the Acupuncture Point LR3 (Taichong)

**DOI:** 10.1155/2020/6760958

**Published:** 2020-04-14

**Authors:** Kanae Umemoto, Munekazu Naito, Naoyuki Hatayama, Shuichi Hirai, Kou Sakabe

**Affiliations:** ^1^Department of Anatomy, Division of Basic Medical Science, Tokai University School of Medicine, 143 Shimokasuya, Isehara, Kanagawa 259-1193, Japan; ^2^Department of Anatomy, Aichi Medical University School of Medicine, 1-1 Yazakokarimata, Nagakute, Aichi 480-1195, Japan

## Abstract

Cutaneous nerves have vascular branches (VBs) that reach the arteries and are thought to be involved in arterial constriction. We aimed to examine the anatomical and histological relationship between the VBs of a cutaneous nerve in the foot and the acupuncture point LR3 (Taichong), which is a depression between the base of the first and second metatarsal bones on the dorsum of the foot and is a source point of the foot. We examined 40 cadaver feet to assess the distribution areas of the VBs of the medial branch of the deep peroneal nerve (MBDPN). MBDPNs were distally followed to identify the point where the VBs reached the arteries. The distance between the point and LR3 was measured. Sympathetic fibers in the VBs were histologically observed using tyrosine hydroxylase (TH) immunostaining. The VBs of the MBDPNs reaching the dorsal pedis arteries were observed in all specimens (100%). The mean distance between LR3 and the point where the VBs of the MBDPN reached the arteries was 3.2 ± 2.6 mm. Among the VBs, 70% were distributed proximal to LR3. Moreover, TH-positive fibers were present in the VBs. These findings revealed that a part of the MBDPN distributed the dorsal pedis artery and contained sympathetic fibers. We also found that the distribution area of the VBs was close to LR3. Our study provides anatomical evidence that LR3 is a specific area and its stimulation would be useful for treating peripheral circulatory failure.

## 1. Introduction

Most cutaneous nerves are distributed to the skin and mainly transmit sensation, while some cutaneous nerves reach the arteries. The nerves reaching the arteries are called vascular branches (VBs), vascular nerves, or arterial branches [1–5]. Balogh et al. reported that sympathetic fibers are present in the VBs of the palmar cutaneous branch of the ulnar nerve [5], suggesting that VBs contribute to arterial constriction.

**Figure 1:**
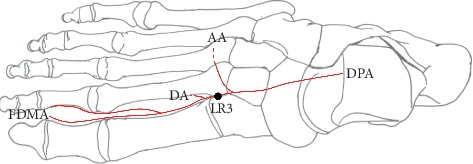
Schematic of the acupuncture point LR3 (Taichong) and the dorsal pedis artery (DPA) on the dorsal side. The dorsal pedis artery (DPA) is a continuation of the anterior tibial artery (ATA) and runs along the dorsum of the foot until the 1st intermetatarsal space. The DPA gives rise to the arcuate, deep plantar, and first dorsal metatarsal arteries on the dorsum of the foot. LR3, acupuncture point “Taichong”; DPA, dorsal pedis artery; AA, arcuate artery; DA, deep planter artery; FDMA, first dorsal metatarsal artery. The black circle indicates acupuncture point LR3.

We previously reported that the distribution areas of the VBs of the superficial branch of the radial nerve were more limited than those of the cutaneous nerves of the forearm and hand. The distribution area is located at a depression between the base of the first and second metacarpal bones on the dorsum of the hand, i.e., at the acupuncture point LI4 [6, 7]. By contrast, the acupuncture point LR3 (Taichong) is close to the dorsal pedis artery (DPA) and located between the first and second metatarsal bones in the depression distal to the junction of the bases of the two bones on the dorsum of the foot [8] and in the area innervated by the medial branch of the deep peroneal nerve (MBDPN) (Figure 1).

Therefore, we hypothesized that the VBs of the MBDPN reach the DPA and that the distribution areas of the VBs are limited to the acupuncture point LR3, which is a source point. The aim of this study was to investigate the anatomical and histological relationship between VBs of the MBDPN and LR3.

## 2. Materials and Methods

### 2.1. Subjects

We examined 20 human cadavers (9 men and 11 women), of which 18 were donated to Tokai University School of Medicine in 2018 and the rest were donated to Aichi Medical University School of Medicine in 2019. Before their passing, the donors gave their informed consent to donate their bodies for clinical research. The format of the document is in accordance with the Japanese law, “Act on Body Donation for Medical and Dental Education”. The Ethics Committee in Tokai University School of Medicine (approval number: 18R-015) and Aichi Medical University School of Medicine (approval number: 2019-121) approved this study. Cadavers with vascular grafts or fixed flexion in the hands and forearms were excluded because we could not confirm the accurate pathway and distribution of the VBs. The distribution areas of the VBs of the MBDPN were examined in all 40 cadaver feet. The mean age of the cadavers was 86.6 ± 9.0 years (range, 63–102 years; men, 86.2 ± 7.5 years; women, 86.8 ± 10.4 years). All cadavers were embalmed using 10% formaldehyde solution.

### 2.2. Anatomical Observation

After the extensor hallucis brevis and tendon of the extensor hallucis longus muscle were reversed, the MBDPN was identified and followed distally to identify the point where the VBs reached the DPA. LR3 was identified at a depression between the base of the first and second metatarsal bones on the dorsum of the foot. The distance between the point where the VBs reached the DPA and LR3 was measured.

All these measurements were performed three times using digital calipers and were measured to the nearest 0.1 mm. The lengths and diameters of the VBs were also measured.

### 2.3. Hematoxylin and Eosin Staining and Tyrosine Hydroxylase Immunostaining

After the anatomical observation, the tissue samples near the point where the VBs reached the DPA were stained with hematoxylin and eosin (HE) and immunostained with tyrosine hydroxylase (TH).

VBs that reached the DPA were obtained from dissected human cadaver feet. The collected samples were fixed in 10% neutral buffered formalin (4% formaldehyde in phosphate-buffered saline (PBS) solution) and processed as per the standard protocol. Paraffin embedding of the collected samples was performed after dehydrating the fixed tissue in ascending grades of alcohol solution. Xylene, which is miscible in both alcohol and paraffin, was used to remove the alcohol. The blocks were made using melted paraffin that was allowed to cool. Once hardened, they were cut to an appropriate size. The tissue samples were sectioned using a rotary microtome. Every 5th section (3-*μ*m) was obtained and subjected to HE staining and TH immunostaining, and 45-*μ*m lengths of VBs were observed along the blood vessel. TH immunostaining was used to detect sympathetic fibers [5].

In the HE staining, the samples were immersed in xylene and alcohol, stained with hematoxylin for 15 min, stained with eosin for 1 min, and then reimmersed in alcohol and xylene.

In the TH immunostaining, the samples were immersed in xylene and alcohol and placed in a fresh 0.3% hydrogen peroxide solution in methanol for 15 min at room temperature (range, 24°C–26°C) to block endogenous peroxidase activity. After a brief rinse in 10% PBS, the samples were placed in a blocking solution (PBS containing 5% normal goat serum) for 10 min at room temperature and incubated at 4°C overnight with rabbit polyclonal anti-TH antibody (1 : 200; AB152, Santa Millipore). After a second brief rinse in 10% PBS, the samples were placed in the blocking solution for 5 min at room temperature and incubated for 40 min at room temperature with a goat anti-rabbit IgG antibody (Number 424141, Nichirei Biosciences Inc.). After a third brief rinse in 10% PBS, the samples were placed in a diaminobenzidine (DAB) solution containing hydrogen peroxide, Tris HCl, and sodium azide for 2 min at room temperature, stained with hematoxylin for 5 min, and then immersed in alcohol and xylene.

The slides were mounted using a synthetic resin. Areas along the blood vessels were observed and photographed with an Olympus BX63 automated microscope (Olympus, Tokyo, Japan), PlanApoN × 2/0.08 objective lens (Olympus, Tokyo, Japan), UPlanSApo × 4/0.16 objective lens (Olympus, Tokyo, Japan), UPlanSApo × 10/0.40 objective lens (Olympus, Tokyo, Japan), UPlanSApo × 20/0.75 objective lens (Olympus, Tokyo, Japan), and Olympus DP73 CCD digital camera (Olympus, Tokyo, Japan).

### 2.4. Statistical Analysis

Results are presented as mean ± standard deviation. Statistically significant differences between the right and left sides of the MBDPN in terms of the distance from LR3 and the length and diameter of the VBs were evaluated using the Mann–Whitney *U* test. Statistical analysis was completed using GraphPad Prism (GraphPad Software Inc., San Diego, CA, USA). The level of statistical significance was set at *P* < 0.05.

## 3. Results

### 3.1. Dissection of the VBs

**Figure 2:**
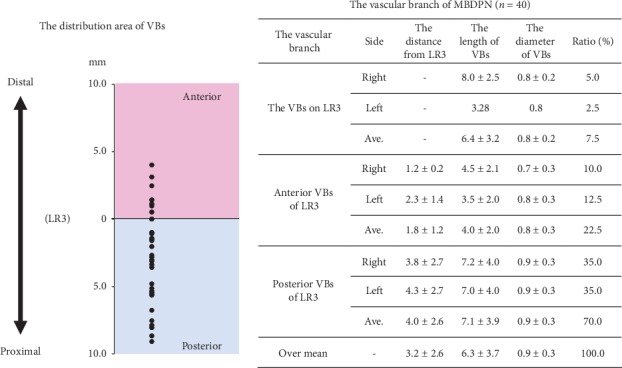
Vascular branches (VBs) of the deep peroneal nerve (MBDPN).

The deep peroneal nerve extended along the anterior tibial artery on the ulnar side and branched into medial and lateral branches. The DPA, which is a continuation of the anterior tibial artery after it crosses the ankle joint, ran over the dorsum of the foot and across the tarsal bones and then ran inferiorly into the first dorsal interosseous muscle, heading toward the plantar aspect of the foot, i.e., the DPA ran over LR3 in all specimens (100%). The VBs of the MBDPN were distributed in the DPA in all specimens (100%). The mean distance between LR3 and the point where the VBs reached the artery was 3.2 ± 2.6 mm (range, 0.0–9.1 mm). The mean length and diameter of the VBs were 6.3 ± 3.7 mm and 0.9 ± 0.3 mm, respectively. VBs in 3 of the 40 feet (7.5%) were distributed only at LR3 (VBs on LR3), VBs in 9 feet (22.5%) were distributed distal to LR3 (anterior VBs of LR3), and other VBs (70.0%) were distributed proximal to LR3 (posterior VBs of LR3) (Figure 2). The mean distances between the LR3 and the point where the VBs distal to the LR3 and proximal to the LR3 reached the artery were 1.8 ± 1.2 mm and 4.0 ± 2.6 mm, respectively. There was no significant difference in the variance between the right and left sides of the MBDPN in the distance from LR3 and the length and diameter of the VBs.

The VBs were distributed in the DPA in all specimens (100%). The mean distance from LR3 to the points where the VBs reached the DPA was 3.2 ± 2.6 mm (range, 0–9.1 mm). VBs in 3 of the 40 cadaver feet (7.5%) were distributed only at LR3 (VBs on LR3), VBs in 9 feet (22.5%) were distributed distal to LR3 (anterior VBs of LR3), and other VBs (70.0%) were distributed proximal to LR3 (posterior VBs of LR3).

**Figure 3:**
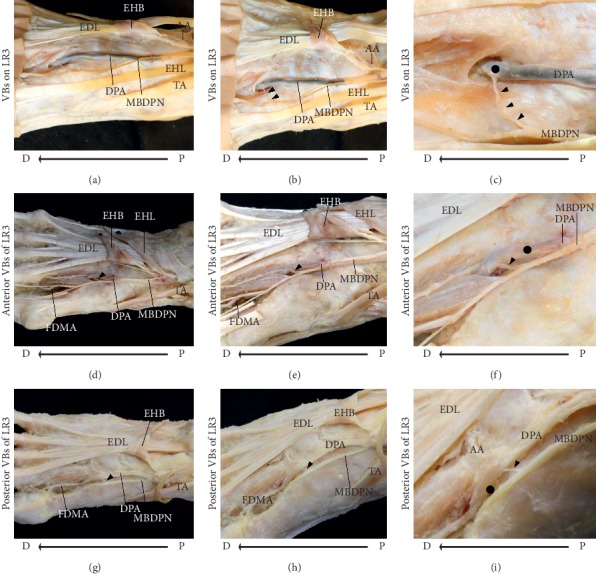
Dissection photographs showing the vascular branches (VBs) of the medial branch of the deep peroneal nerve (MBDPN). Dissection photographs showing the VBs of the MBDPN on the right side. Case 1 (a)–(c). (a) Dissection photograph of the radial side after removal of the skin and subcutaneous tissue. (b) Dissection photograph of the dorsal side after removal of the skin and subcutaneous tissue. (c) Enlarged dissection photograph of (b). Case 2 (d)–(f). (d) Dissection photograph of the dorsal side after removal of the skin and subcutaneous tissue. (e) Dissection photograph of the radial side after removal of the skin and subcutaneous tissue. (f) Enlarged dissection photograph of (e). Case 3 (g)–(i). (g) Dissection photograph of the dorsal side after removal of the skin and subcutaneous tissue. (h) Dissection photograph of the radial side after removal of the skin and subcutaneous tissue. (i) Enlarged dissection photograph of (h). MBDPN, the medial branch of deep peroneal nerve; DPA, the dorsal pedis artery; AA, the arcuate artery; FDMA, the first dorsal metatarsal artery; EDL, the tendon of the extensor digitorum longus muscle; EHB, the extensor hallucis brevis muscle; EHL, the tendon of the extensor hallucis longus muscle; TA, the tendon of the tibialis anterior muscle; P, proximal; D, distal.

In case 1, the *VBs were located directly on LR3 (VBs on LR3)*. The cadaver was that of a 94-year-old Japanese woman who died from senescence (Figures 3(a)–3(c)). After the extensor hallucis brevis muscle was reversed, the MBDPN running along the DPA on the radial side was observed. The DPA gave rise to the arcuate artery and extended inferiorly into the first dorsal interosseous muscle. The MBDPN branched into VBs and a cutaneous branch, and the VBs were distributed at LR3. The length and diameter of the VBs were 9.71 and 0.98 mm, respectively.

The black arrowheads indicate the vascular branches (VBs), and orange arrows indicate acupuncture point LR3.

In case 2, the *VBs were distal to LR3 (anterior VBs of the LR3)*. The cadaver was that of an 81-year-old Japanese man who died of Hodgkin's disease (Figures 3(d)–3(f)). After the extensor hallucis brevis and tendon of the extensor hallucis longus muscles were reversed, the MBDPN running along the DPA on the radial side was observed. After branching below the first dorsal interosseous muscle, the DPA gave rise to the first dorsal metatarsal artery, which ran distally on the first dorsal interosseous muscle. The MBDPN branched into VBs and a cutaneous branch, and the distal distance between LR3 and the point where the VBs reached the artery was 0.52 mm. The length and diameter of the VBs were 3.35 and 0.78 mm, respectively.

In case 3, the VBs were proximal to LR3 (posterior VBs *of LR3*). The cadaver was that of an 88-year-old Japanese woman who died of pancreatic cancer (Figures 3(g)–3(i)). After the extensor hallucis brevis and tendon of the extensor hallucis longus muscle were reversed, the MBDPN running along the DPA on the radial side was observed. After giving rise to the arcuate artery, the DPA ran inferiorly into the first dorsal interosseous muscle and gave rise to the first dorsal metatarsal artery, which ran distally on the first dorsal interosseous muscle. The MBDPN branched into VBs and a cutaneous branch, and the proximal distance between LR3 and the point where the VBs reached the artery was 3.03 mm. The length and diameter of the VBs were 4.47 and 0.9 mm, respectively.

### 3.2. HE Staining and TH Immunostaining

**Figure 4:**
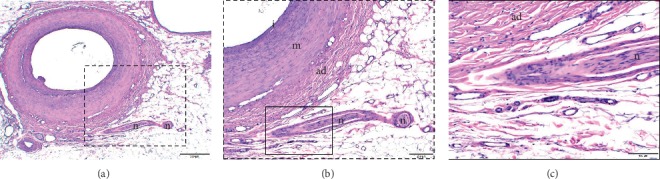
Vascular branches (VBs) of the medial branch of the deep peroneal nerve (hematoxylin and eosin stain). (a) The nerve fibers of the VBs of the medial branch of the deep peroneal nerve seen close and extending to the dorsal pedis artery (DPA) and entering into the adventitia of the DPA (hematoxylin and eosin stain); scale bar indicates 500 *μ*m (×2/0.08 objective lens). (b) Enlarged photograph of the dotted rectangle in (a); scale bar indicates 200 *μ*m (×4/0.16 objective lens). (c) Enlarged photograph of the dotted rectangle in (b); scale bar indicates 100 *μ*m (×10/0.40 objective lens). ad, adventitia; I, intima; m, media; n, nerve fibers of the VBs.

**Figure 5:**
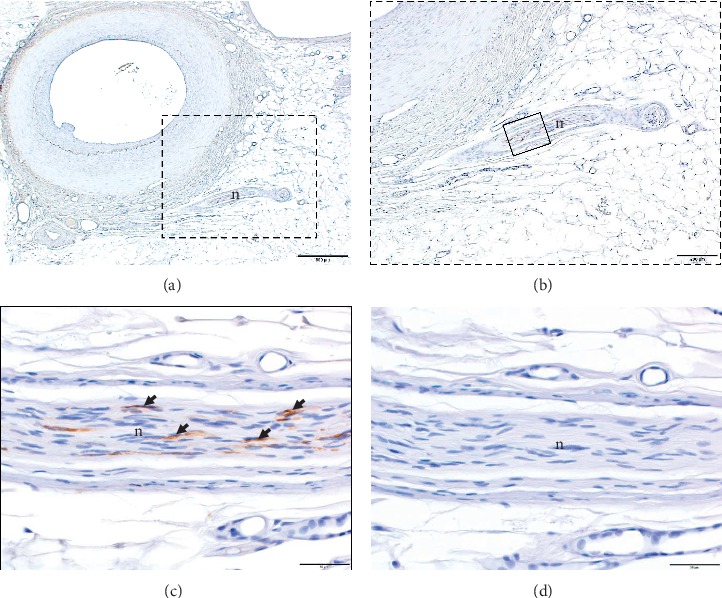
Vascular branches (VBs) of the medial branch of the deep peroneal nerve (tyrosine hydroxylase immunohistochemistry). (a)–(c) Nerve fibers of the VBs adjacent to the dorsal pedis artery. Sympathetic fibers were present in the VBs. Brown dots are sympathetic fibers (tyrosine hydroxylase immunohistochemistry). (b) Enlarged photograph of the dotted rectangle in (a). (c) Enlarged photograph of the dotted rectangle in (b). Black arrowheads also indicate the sympathetic fibers. (d) Negative control shows the absence of tyrosine hydroxylase antibodies. n, nerve fibers of the VBs. (a) Scale bar indicates 500 *μ*m (×2/0.08 objective lens). (b) Scale bar indicates 200 *μ*m (×4/0.16 objective lens). (c) and (d) Scale bars indicate 50 *μ*m (×20/0.75 objective lens).

The nerve fibers of the VBs coursed along the DPA (HE staining); the VBs ran increasingly closer to the DPA (Figures 4(a)–4(c)). Moreover, sympathetic fibers were present in the VBs (TH immunostaining, Figures 5(a)–5(c)).

## 4. Discussion

Our findings first revealed that a part of the MBDPN reached the DPA and contained sympathetic fibers. Furthermore, we found that the distribution area of the VBs was limited at the acupuncture point LR3 (Taichong).

We previously showed that the distribution areas of parts of the cutaneous branches of the radial nerve, i.e., VBs, were limited at the radial artery [6]. By contrast, several studies have reported that VBs reach the femoral, popliteal, anterior tibial, posterior tibial, and peroneal arteries [9–13]. However, it was not clear whether the VBs of the cutaneous branch reached the DPA. In this study, the VBs of the MBDPN were distributed in the DPA in all specimens (100%, Figure 2), and the histological observation showed that they consisted of sympathetic fibers and were close to the DPA (Figures 4 and 5). Therefore, the VBs of the MBDPN may regulate the blood flow of the DPA.

LR3 is in the Jueyin Liver Meridian of the Foot, a special acupuncture source point that has been used to relieve stress, headache, dizziness, and eyestrain. Stimulation of LR3 specifically activates areas in the brain [14], alters cerebral glucose metabolism in the hypothalamus [15], increases blood flow in the skin [16], and is used for treating peripheral circulatory failure in the foot, i.e., Raynaud's phenomenon [17, 18]. According to Omole et al., stimulation of LI4 improves pain severity, joint stiffness, and the color of the fingers and toes [19]. In the study of the positional relationship between the acupuncture points in the Yangming Large Intestine Meridian of the Hand and the VBs from the superficial branch of the radial nerve (cutaneous nerve), LI4 was significantly closer to the Yangming Large Intestine Meridian of Hand than the other acupuncture points [7]. In our study, the VBs of the MBDPN were distributed at the area close to LR3 (3.2 ± 2.6 mm) (Figure 2). Thus, stimulation of an area up to 3.2 ± 2.6 mm of LR3 may be effective for treating Raynaud's phenomenon. Among the VBs, 70% were distributed posterior to LR3 and their mean length was 7.1 ± 3.9 mm (Figure 2). Transcutaneous electrical nerve stimulation, which is a noninvasive peripheral stimulation technique, of an area up to 7.1 ± 3.9 mm proximal to LR3 may also be effective for treating Raynaud's phenomenon.

Periarterial sympathectomy, a surgical procedure in which the adventitia of the artery, where the VBs innervate is stripped off, is used for treating Raynaud's phenomenon. The procedure targets VBs [20, 21] and strips the adventitia to increase blood flow [22, 23]. In our previous report, we found that the distribution areas of the VBs of the superficial branch of the radial nerve were limited at the acupuncture point LI4 [7]. Therefore, we presumed that LI4 is a suitable site for stripping off the adventitia in periarterial sympathectomy and that its stimulation can improve Raynaud's phenomenon [7]. Meanwhile, other studies have shown that periarterial sympathectomy for the DPA can be used to treat Raynaud's phenomenon in the foot [24, 25]. In this study, we revealed that the distribution areas of the VBs of MBDPN were limited at the acupuncture point LR3, and there was no significant difference in the variance between the right and left sides of the MBDPN in terms of the distance from LR3. Our findings suggest that an area around LR3, measuring up to 3.2 ± 2.6 mm, could be a suitable site for periarterial sympathectomy in both feet.

LR3 and LI4 are source points, which are points in the body that react when one or more of the five viscera are affected; therefore, these acupuncture points are extensively used in the clinical setting [26]. These points are further divided into four points (left LR3, right LR3, left LI4, and right LI4), referred to as the “Four Gates” (Siguan points), which play a role in activating the energy flow by opening the four gates of vital energy [27]. Therefore, LR3 and LI4 have been considered as special anatomical sites.

## 5. Conclusion

In conclusion, we determined that the acupuncture point of LR3, especially the proximal LR3, is close to the VBs of MBDPN; we also found that sympathetic fibers are present in the VBs of MBDPN.

Our findings provide anatomical evidence that the area of LR3 is a specific area and stimulation of LR3 can be used to treat peripheral circulatory failure.

## Data Availability

The data used to support the findings of this study are available from the corresponding author upon request.

## References

[1] Henle J. (1868). *Handbuch der Nervenlehre Des Menschen*.

[2] Kramer J. G., Todd T. W. (1914). The distribution of nerves to the arteries of the arm, with a discussion of the clinical values of results. *The Anatomical Record*.

[3] Pick J. (1958). The innervation of the arteries in the upper limb of man. *The Anatomical Record*.

[4] McCabe S. J., Kleinert J. M. (1990). The nerve of Henlé. *The Journal of Hand Surgery*.

[5] Balogh B., Valencak J., Vesely M. (1999). The nerve of Henle: an anatomic and immunohistochemical study. *The Journal of Hand Surgery (A)*.

[6] Umemoto K., Ohmichi M., Ohmichi Y. (2018). Vascular branches from cutaneous nerve of the forearm and hand: application to better understanding raynaud’s disease. *Clinical Anatomy*.

[7] Umemoto K., Naito M., Tano K. (2019). Acupuncture point “Hegu” (LI4) is close to the vascular branch from the superficial branch of the radial nerve. *Evid Based Complement Alternat Med*.

[8] World Health Organization (2008). *WHO Standard Acupuncture Point Locations in the Western Pacific Region*.

[9] Potts L. W. (1914). The distribution of nerves to the arteries of the leg. *Anatomischer Anzeiger*.

[10] Blair D. M., Duff D., Bingham J. A. (1930). The anatomical result of peri-arterial sympathectomy. *British Journal of Surgery*.

[11] Coates A. E. (1932). Observations on the distribution of the arterial branches of the peripheral nerves. *Journal of Anatomy*.

[12] Wilde F. R. (1951). The perivascular neural pattern of the femoral region. *British Journal of Surgery*.

[13] Mustalish A. C., Pick J. (1964). On the innervation of the blood vessels in the human foot. *The Anatomical Record*.

[14] Zheng Y., Wang Y., Lan Y. (2016). Imaging of brain function based on the analysis of functional connectivity - imaging analysis of brain function by fmri after acupuncture. *African Journal of Traditional, Complementary and Alternative Medicines*.

[15] Li J., Wang Y., He K. (2018). Effect of acupuncture at LR3 on cerebral glucose metabolism in a rat model of hypertension: a ^18^F-FDG-PET study. *Evidence-Based Complementary and Alternative Medicine*.

[16] Nishimaki N., Yamada N., Ikeuchi T., Shinohara S., Mathumoto T., Mori K. (1985). Changes in the peripheral circulation in the lower extremity caused by moxibustion-measurements of skin and deep tissue temperature-. *The Bulletin of Meiji College of Oriental Medicine*.

[17] Bao J. Z. (1988). Acupuncture treatment of Raynaud’s disease–a report of 43 cases. *Journal of Traditional Chinese Medicine*.

[18] Appiah R., Hiller S., Caspary L., Alexande K., Creutzig A. (1997). Treatment of primary Raynaud’s syndrome with traditional Chinese acupuncture. *Journal of Internal Medicine*.

[19] Omole F. S., Lin J. S., Chu T., Sow C. M., Flood A., Powell M. D. (2012). Raynaud’s phenomenon, cytokines and acupuncture: a case report. *Acupuncture in Medicine*.

[20] El-Gammal T. A., Blair W. F. (1991). Digital periarterial sympathectomy for ischaemic digital pain and ulcers. *Journal of Hand Surgery*.

[21] Chiou G., Crowe C., Suarez P., Chung L., Curtin C., Chang J. (2015). Digital sympathectomy in patients with scleroderma. *Annals of Plastic Surgery*.

[22] Morgan R. F., Wilgis E. F. S. (1986). Thermal changes in a rabbit ear model after sympathectomy. *The Journal of Hand Surgery*.

[23] Pollock D. C., Li Z., Rosencrance E., Krome J., Koman L. A., Smith T. L. (1997). Acute effects of periarterial sympathectomy on the cutaneous microcirculation. *Journal of Orthopaedic Research*.

[24] Li Z., Smith B., Holden M., Koman L. (2009). Periarterial sympathectomy of the foot for the treatment of necrotizing Raynaud’s phenomena. *Journal of Reconstructive Microsurgery*.

[25] Singh S., Bukhari M. (2016). Periarterial digital sympathectomy for severe ischaemia of Raynaud’s. *Clinics of Surgery*.

[26] Endo J. (1986). Significance of source points. *Kampo Medicine*.

[27] Chang X., Chang K.  (1987). Chinese Acupuncture and Moxibustion.

